# General Analytical Procedure for Determination of Acidity Parameters of Weak Acids and Bases

**DOI:** 10.1155/2015/530731

**Published:** 2015-01-26

**Authors:** Bogusław Pilarski, Roman Kaliszan, Dariusz Wyrzykowski, Janusz Młodzianowski, Agata Balińska

**Affiliations:** ^1^Cerko Sp. z o.o. Sp.K, Al. Zwycięstwa 96/98, 81-451 Gdynia, Poland; ^2^Department of Biopharmaceutics and Pharmacodynamics, Medical University of Gdańsk, Gen. J. Hallera 107, 80-416 Gdańsk, Poland; ^3^Faculty of Chemistry, University of Gdańsk, Wita Stwosza 63, 80-308 Gdańsk, Poland

## Abstract

The paper presents a new convenient, inexpensive, and reagent-saving general methodology for the determination of p*K*_a_ values for components of the mixture of diverse chemical classes weak organic acids and bases in water solution, without the need to separate individual analytes. The data obtained from simple pH-metric microtitrations are numerically processed into reliable p*K*_a_ values for each component of the mixture. Excellent agreement has been obtained between the determined p*K*_a_ values and the reference literature data for compounds studied.

## 1. Introduction

Prediction or determination of p*K*_a_ value is of great importance in chemistry, in particular in life and material sciences, pharmaceutical industry, and other R&D oriented enterprises. Important drug properties, such as lipophilicity, solubility, and transmembrane transfer, are all pH dependent. Also, rational drug formulation requires the knowledge of p*K*_a_. The proportion of drugs with an ionizable group has been estimated at 95% [1], but only 62.9% of drugs under analysis were ionizable at pH 2–12 [2]. According to Wells data 75% of drugs are weak bases and 20% weak acids and the remaining contain nonionics, ampholytes, and alcohols [1].

Recently, some theoretical approaches were employed to predict the p*K*_a_ value, for example,* ab initio* quantum mechanical calculations [3, 4] or QSPR (quantitative structure-property relationship) modeling [5, 6] as well as QSPR models which employ partial atomic charges as descriptors [7, 8]. The theoretical models take into account electronic effects (induction, resonance), solvation of compounds of type HA, BH and their ionic forms, that is, A^−^ and BH^+^, hydrogen bonding, and various stereochemical effects.

This report presents an application of pH-metric microtitration to determine standard p*K*_a_ parameters of components of mixtures of various weak acids and bases by employing a technologically advanced potentiometer device and a software based on an algorithm straightforwardly accounting for complex acid-base equilibria (see below). A composition of the mixtures under study can be expressed as follows:

(1) H3A1+H2A2+HA3+B1+B2+R1R2CH2    +R1R2N–H+Ar-OH

where H_3_A_1_ + H_2_A_2_ + HA_3_ represents 3-H, 2-H, and 1-H protic carboxylic acids, B_1_ + B_2_ represents organic bases, (R_1_R_2_)CH_2_ represents the so-called C–H acids, R_1_R_2_N–H– represents the N–H acids, and ArOH denotes phenolic/enolic moiety (O–H acids). The C–H, N–H, and O–H acids are often reported as tautomeric forms of heterocyclic compounds with pharmacological activity and are identified within different groups of natural compounds (flavonoids, quinines, etc.).

*Numerical Modelling*. Numerical procedures are based on an original algorithm elaborated by Kostrowicki and Liwo [9] as well as the* CVEQUID* program, which was adopted in the Cerko Lab software within the Cerko Lab System microtitrator unit (Cerko, Gdynia, Poland). All details concerning Kostrowicki and Liwo algorithm were described previously [10]. The* CVEQUID* program is based on a least-square method for the determination of all parameters and takes into account all the sources of experimental errors considered in potentiometry, that is,

- electrode calibration parameters (*E* ^0^, the standard potential (cell constant) and *S*, the standard Nernstian slope parameter);
- composition of titrand D, its concentration *C*_0_ (mol/L), and volume *V*_0_ (mL);
- composition of titrant T, its concentration (mol/L), and added volume (mL);
- measured EMF (the electromotive force) in mV.

Within the Cerko Lab System software, the equilibrium is denoted as* model*. The* model* consists of a set of equations. Each equation is related to a particular p*K* value and to p*K*_w_. The* model* includes also information about the composition of titrant T and titrand D. The stoichiometric matrix, required for the numerical procedures, is generated from the model automatically. The representative models and the corresponding stoichiometric matrix for H_2_A (model 1) as well as H_3_A + H_2_A_1_ + HA_2_ (model 2) systems are given below.

*Model 1*. Reagents include titrand D = H_2_A and titrant T = OH. Individual equilibria that contribute to the overall equilibrium of the system are as follows:

1. H_2_A = H^+1^ + HA^−1^,  *K*_1_ = [H^+1^][HA^−1^]/[H_2_A],
2. HA^−1^ = H^+^ + A^−2^,  *K*_2_ = [H^+1^][A^−2^]/[HA^−1^],
3. 0 = H^+1^ + OH^−1^,  *K*_w_ = *K*[H_2_O] = [H^+1^][OH^−1^].

The stoichiometric matrix for the above model is presented in Table 1.

The concentration of titrand, D (*C*_0_ = *C*_01_ + *C*_02_ + *C*_03_⋯), and titrand volume (*V*_0_ = *V*_01_+ *V*_02_ + *V*_03_⋯), used in the potentiometric titration, result from mixing of various types of acid-base solutions.

*Model 2*. Reagents include titrand D = H_3_A + H_2_A_1_ + HA_2_ and titrant T = OH (Table 2). The model consists of a water solution of different types of acids. The equilibrium constants and stoichiometric matrix are as follows:

1. H_3_A = H^+1^ + H_2_A^−1^,  *K*_1A_ = [H^+1^][H_2_A^−1^]/[H_3_A],
2. H_2_A^−1^ = H^+1^ + HA^−2^,  *K*_2A_ = [H^+1^][HA^−2^]/[H_2_A^−1^],
3. HA^−2^ = H^+^ + A^−3^,  *K*_3A_ = [H^+1^][A^−3^]/[HA^−2^],
4. H_2_A_1_ = H^+1^ + HA_1_^−1^,  *K*_1A_1__ = [H^+1^][HA_1_^−1^]/[H_2_A_1_],
5. HA_1_^−1^ = H^+^ + A_1_^−2^,  *K*_2A_1__ = [H^+1^][A_1_^−2^]/[HA_1_^−1^],
6. HA_2_ = H^+1^ + A_2_^−1^,  *K*_1A_2__ = [H^+1^][A_2_^−1^]/[HA_2_],
7. 0 = H^+1^ + OH^−1^,  *K*_w_ = *K*[H_2_O] = [H^+1^][OH^−1^].

*General Model. *Reagents include titrand D  =  H_3_A + H_2_A_1_ + HA_2_+ B + B_1_+ (R_1_R_2_)CH_2_ + R_1_R_2_NH+ Ar-OH + *n*HX (HX  as  a  strong  mineral  acid)…, and titrant T = OH. The presence of a strong mineral acid causes transformation of all the basic reagents into an acidic form—conjugate acid of amine.

**Scheme 1:**
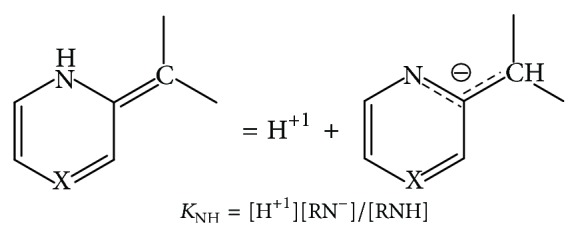


The model consists of a water solution of different types of acids, bases, C–H, N–H, and O–H acids. The existing equilibria in solution are given below:

1. H_3_A = H^+1^ + H_2_A^−1^,  *K*_1A_ = [H^+1^][H_2_A^−1^]/[H_3_A],
2. H_2_A^−1^ = H^+1^ + HA^−2^,  *K*_2A_ = [H^+1^][HA^−2^]/[H_2_A^−1^],
3. HA^−2^ = H^+^ + A^−3^,  *K*_3A_ = [H^+1^][A^−3^]/[HA^−2^],
4. H_2_A_1_ = H^+1^ + HA_1_^−1^,  *K*_1A_1__ = [H^+1^][HA_1_^−1^]/[H_2_A_1_],
5. HA_1_^−1^ = H^+^ + A_1_^−2^,  *K*_2A_1__ = [H^+1^][A_1_^−2^]/[HA_1_^−1^],
6. HA_2_ = H^+1^ + A_2_^−1^,  *K*_1A_2__ = [H^+1^][A_2_^−1^]/[HA_2_],
7. BH^+1^ = H^+1^ + B,  *K*_BH^+1^_ = [H^+1^][B]/[BH^+1^],
8. (R_1_R_2_)CH_2_ = H^+1^ + R_1_R_2_CH^−1^,  *K*_(R_1_R_2_)CH_2__ = [H^+1^][(R_1_R_2_)CH^−1^]/[(R_1_R_2_)CH_2_],
9. see Scheme 1,
10. ArOH = H^+1^ + ArO^−1^,  *K*_OH_ = [H^+1^][ArO^−1^]/[ArOH],
11. H_2_O = H^+1^ + OH^−1^,  *K*_w_ = *K*[H_2_O] = [H^+1^][OH^−1^].

## 2. Experimental

### 2.1. Apparatus and Reagents

The pH-metric titrations were performed in a 30 mL thermostated (25.0 ± 0.2°C) cell, using a Cerko Lab microtitration unit, fitted with a pH electrode (Hydromet ERH-13-6). The temperature was controlled using the Lauda E100 circulation thermostat. The electrode was calibrated with the use of buffer solutions: potassium hydrogen phthalate (pH 4.00), citric acid/Na_2_HPO_4_ (pH 7.00), and boric acid/KCl/NaOH (pH 10.00).

Titrant T (0.1 mol/L NaOH) was standardized according to the general analytical procedure and protected from carbon dioxide. Double distilled water of conductivity approximately 0.18 *μ*S/cm was used throughout for the preparation of aqueous solutions of organic acids and bases under study. It was freshly produced in order to avoid carbon dioxide absorption. Other reagents together with their abbreviations used in the text are listed in Abbreviations section.

### 2.2. Analytical Procedure

Volume *V*_0_ of 4.0 mL to 5.0 mL of titrand (D) was titrated with 0.1 mol·L^−1^ of titrant (T) using a Cerko Lab System, equipped with a syringe pump. Titrant (T) was added to titrand (D) in increments of 0.01 mL, with a pause of 7 s. The p*K*_a_ values were calculated from the experimental data points {(*V*_*j*_, pH_*j*_)∣*j* = 1,…, *N*} according to the Kostrowicki and Liwo algorithm [9, 10].

## 3. Results and Discussion

### 3.1. Carboxylic Acids Mixture

**Figure 1:**
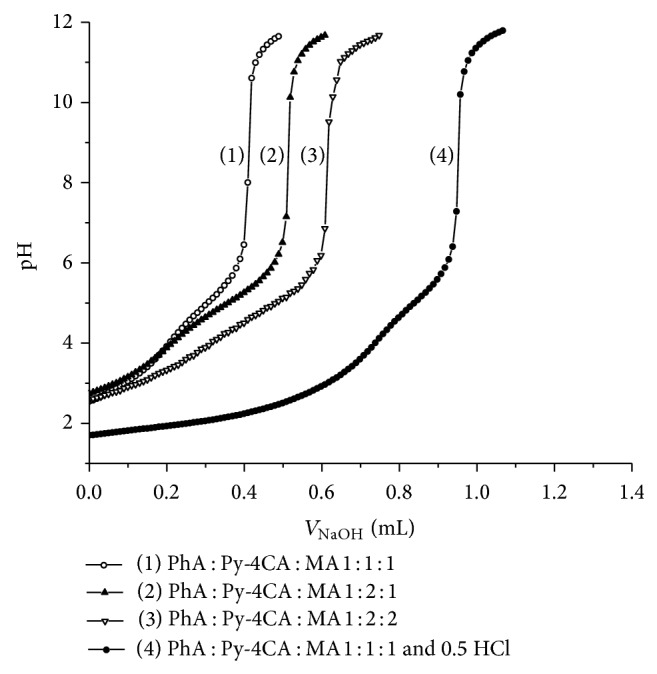
Titration curves pH = *f*(*V*_NaOH_) obtained for the mixture of PhA, Py4CA, and MA with different mole ratio of acids and with excess of HCl.

**Figure 2:**
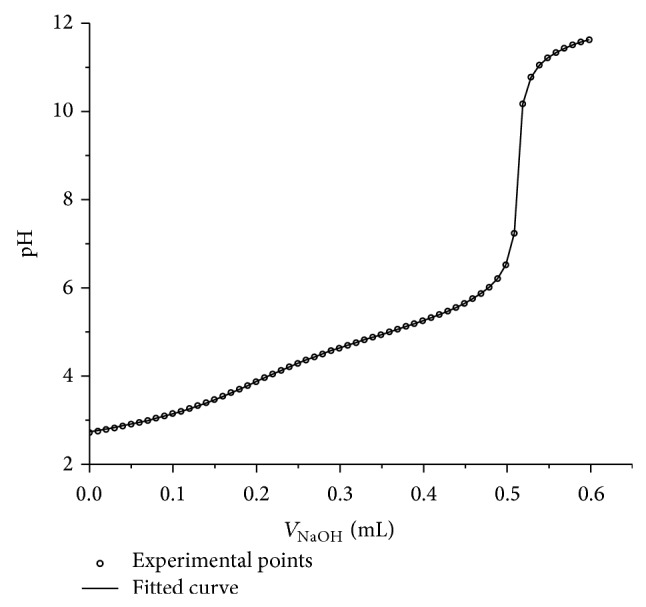
Titration and fitted curves pH = *f*(*V*_NaOH_) obtained for mixture of PhA, Py-4CA, and MA with mole ratio of acids 1 : 2 : 2.

The representative pH titration curves for the mixture of carboxylic acids PhA : Py-4CA : MA are presented in Figures 1 and 2. Compositions of titrand D for the mixture (PhA : Py-4CA : MA) were as follows: 1 : 1 : 1, 1 : 2 : 1, 1 : 2 : 2, and 1 : 1 : 1 with 0.5 mole ratio of HCl. The titration and fitted curves pH = *f*(*V*_NaOH_) obtained for mixture with molar ratio of the components 1 : 2 : 2 (PhA : Py-4CA : MA) are shown in Figure 2. The p*K*_a_ data determined for the mixtures of carboxylic acids under study are listed in Tables 3 and 4.

### 3.2. Organic Bases in Protonated Form (Cationic Acids)

The amino group is one of the most fundamental functional groups considered in organic and pharmaceutical chemistry and its p*K*_a_ is an important and extensively studied property. The p*K*_a_ value of the amino group can vary over several orders of magnitude (ammonia, p*K*_a_ = 9.26; aniline, p*K*_a_ = 4.63 [11]), depending on its chemical environment. In our study the p*K*_a_ refers to the conjugate acid B + H^+1^ = BH^+1^ and dissociation according to the scheme: BH^+1^ = B + H^+1^.

We have tested a mixture of organic bases exemplified by aniline and pyridine derivatives (2- and 4-substituted aminopyridines and methylpyridine) at the presence of equimolar ratio of HCl. Organic bases exist in the system in the protonated form (cationic acids, BH^+1^) and dissociate according to the scheme: BH^+1^ = B + H^+1^, *K*_BH_^+^ = [B][H^+1^]/[BH^+1^].

Based on the titration curve of mixture of amines (presented in the form of cationic acids), the p*K*_a_ values were determined for aniline (B) and for 2- and 4-aminopyridine (2-NH_2_Py, 4-NH_2_Py). The heterocyclic five-membered ring systems of imidazole (Im), benzotriazole (Bt), and benzimidazole (Bi) were also investigated. We have applied a general procedure for the titration of mixtures of different types (and concentration) of organic acids and bases. The elaborated procedure was also tested in the presence of biological buffers exemplified by 2-(N-morpholino) ethane-sulfonic acid (Mes) [12].

The values of dissociation constants obtained for mixtures of different type of bases and acids are listed in Tables 5 and 6.

### 3.3. Mixtures of Amino acids with Organic Acids and Bases

The presented general procedure was applied for studying the system consisting of amino acids, organic acids, and bases. The p*K*_a_ values of this type of mixtures were calculated based on a single titration curve. Experimental results confirm the general application of the proposed procedure for the determination of p*K*_a_ value for mixtures of any degree of complexity composed of weak acids and bases. The p*K*_a_ values of weak acids (H_*n*_A), bases (B), and amino acids (AB^±^) in the mixture of these types of components were determined. Composition of the tested mixtures (titrand D) and p*K*_a_ values are listed in Table 7.

### 3.4. Phenol and Enol OH-Acids as Components of Titrand D

The acidity of the phenol group (OH-acid) depends on the substituent of the aromatic ring and its p*K*_a_ ranges from 4 to 11 [8]. We have performed the titration and relevant calculations for several mixtures of phenolic compounds, exemplified by 4-NO_2_ phenol and a drug N-(4-hydroxyphenyl)acetamide (paracetamol) with different type of organic acids and bases as titrands D. The results are summarised in Table 8.

### 3.5. Heterocyclic N–H-Acids as Components of Titrand D

The barbituric acid (BA) and a new class of 2(1H)-pyrazylidene acetonitrile derivatives (2(1H)PyAN), with marked pharmaceutical importance [13, 14], were tested at the presence of phthalic acid. Barbituric acid was also tested at the presence of different drugs (Table 9).

### 3.6. Determination of p*K*_a_ Values for Different Drugs as a Components of Titrand D

For all tested compounds with pharmaceutical importance we confirmed that the elaborated method could be recommended as a general approach to the determination of p*K*_a_ values for weak acids and bases in mixtures of any degree of complexity. The composition of titrand D and the p*K*_a_ values determined for drugs under study are listed in Table 10.

## 4. Conclusions

**Figure 3:**
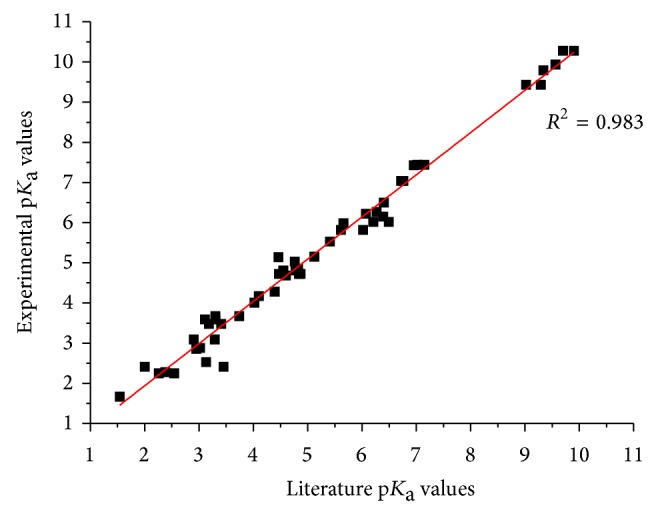
Plot of experimental versus literature p*K*_a_'s values for the compounds under study.

A new approach for studying equilibrium constants for the dissociation of different types of weak electrolytes present in a mixture of any degree of complexity has been proposed. Potentiometric titration technique and numerical procedure based on an original algorithm elaborated by Kostrowicki and Liwo and adopted in the Cerko Lab software have successfully been applied to obtain the p*K*_a_ values of a variety of classes of compounds comprising of common organic acids and bases, amino acids, phenols and enols OH-acids, and heterocyclic N–H-acids as well as compounds of pharmaceutical importance. It was shown that the p*K*_a_ values of the compound present in the mixture can be determined directly without the need to separate individual analytes. The obtained p*K*_a_ values of the electrolytes under study are in a good agreement with those reported in the literature (Table 11, Figure 3). Thus, the presented methodology can be considered as a fast, simple, inexpensive, and reagents-saving way for studying equilibria in the mixture of electrolytes. Moreover, it does not require a highly trained personnel. The methodology described in this paper can be routinely used in a regular analytical practice.

## Figures and Tables

**Table 1:** Stoichiometric matrix for model 1.

	H^+1^	H_2_A	HA^−1^	A^−2^	OH^−1^
p*K*_1_	1	−1	1	0	0
p*K*_2_	1	0	−1	1	0
p*K*_w_	1	0	0	0	1

0 denotes a species that does not take part in equilibrium; −1 donates substrate (left side of equilibria equation); 1 donates product (right side of equilibria equation).

**Table 2:** Stoichiometric matrix for model 2.

	H^+1^	H_3_A	H_2_A^−1^	HA^−2^	A^−3^	H_2_A_1_	HA_1_^−1^	A_1_^−2^	HA_2_	A_2_^−1^	OH^−1^
p*K*_1A_	1	−1	1	0	0	0	0	0	0	0	0
p*K*_2A_	1	0	−1	1	0	0	0	0	0	0	0
p*K*_3A_	1	0	0	−1	1	0	0	0	0	0	0
p*K*_1A_1__	1	0	0	0	0	−1	1	0	0	0	0
p*K*_2A_1__	1	0	0	0	0	0	−1	1	0	0	0
p*K*_1A_2__	1	0	0	0	0	0	0	0	−1	1	0
p*K*_w_	1	0	0	0	0	0	0	0	0	0	1

**Table 3:** The experimental p*K*_a_ data obtained for the mixture of PhA, Py4CA, and MA at various compositions of titrand D.

Composition of D	p*K*_*n*_	p*K*_aPhA_ ± s	p*K*_aPy4CA_ ± s	p*K*_aMA_ ± *s*
PhA + Py4CA + MA (1 : 1 : 1)	p*K*_1_	2.97 ± 0.07	—	3.30 ± 0.03
	p*K*_2_	5.31 ± 0.04	4.69 ± 0.03	—

PhA + Py4CA + MA (1 : 2 : 2)	p*K*_1_	2.81 ± 0.06	—	3.12 ± 0.03
	p*K*_2_	5.45 ± 0.03	4.71 ± 0.01	—

PhA + Py4CA + MA (1 : 2 : 1)	p*K*_1_	2.78 ± 0.04	4.76 ± 0.02	3.4^*^
	p*K*_2_	5.53 ± 0.04		

^*^p*K*_a_ value const. taken from the literature [see Table 11].

**Table 4:** The experimental p*K*_a_ data obtained for the mixture of A + MA + PhA + Py3CA and 2,6PyDCA + Py3CA.

Composition of D	p*K*_*n*_	p*K*_aPhA_ ± *s*	p*K*_aPy3CA_ ± s	p*K*_aMA_ ± *s*	p*K*_aA_ ± *s*
A + MA + PhA + Py3CA	p*K*_1_	3.11 ± 0.05	—	3.74 ± 0.03	4.68 ± 0.02
	p*K*_2_	5.62 ± 0.03	4.82^*^	—	—

Composition of D	p*K*_*n*_	p*K*_a2,6PyDCA_ ± *s*	p*K*_aPy3CA_ ± *s*	—	—

2,6PyDCA + Py3CA	p*K*_1_	2.41 ± 0.04	—	—	—
	p*K*_2_	4.72 ± 0.02	5.19 ± 0.04	—	—

Composition of D	p*K*_*n*_	p*K*_aPhA_ ± *s*	p*K*_aPy3CA_ ± *s*	p*K*_aCA_ ± *s*	—

PhA + Py3CA + CA	p*K*_1_	3.12 ± 0.03	—	3.04 ± 0.03	—
	p*K*_2_	5.53 ± 0.03	4.82^*^	4.46 ± 0.05	—
	p*K*_3_	—	—	6.05 ± 0.04	—

^*^p*K*_a_ value const. from the literature [see Table 11].

**Table 5:** The p*K*_a_'s values of compounds determined in the multicomponent mixture of amines, heterocyclic moiety, and weak (Mes) and strong (HCl) acids at 25°C.

Composition of D	p*K*_A_ ± *s *	p*K*_4NH_2_Py_ ± *s *	p*K*_2NH_2_Py_ ± *s *	p*K*_FA_ ± *s *
A+ HCl	4.650 ± 0.02	—	—	—
A + 4NH_2_Py + HCl	4.53 ± 0.04	9.27 ± 0.18	—	—
A + 4NH_2_Py + 2NH_2_Py + HCl	5.13 ± 0.07	9.59 ± 0.07	7.18 ± 0.07	—
A + 2NH_2_Py + FA	4.7^*^	—	6.7^*^	3.05 ± 0.04
				4.26 ± 0.02

Composition of D	p*K*_Mes_ ± *s *	p*K*_2NH_2_Py_ ± *s *	p*K*_Py3CA_ ± *s *	p*K*_Bt_ ± *s *
			or p*K*_Py4CA_ ± *s *	or p*K*_Bi_ ± *s *

Mes + Bt	6.24 ± 0.05	—	—	8.75 ± 0.59
Mes + 2NH_2_Py + Bi + HCl	6.28 ± 0.05	7.22 ± 0.05	—	5.36 ± 0.04
Mes + 2NH_2_Py + Py3CA	6.29 ± 0.03	7.22 ± 0.03	4.78 ± 0.03	—
Mes + 2NH_2_Py + Py4CA	6.29 ± 0.34	6.39 ± 0.34	4.76 ± 0.30	—

Composition of D	p*K*_ASA_ ± *s *	p*K*_2NH_2_Py_ ± *s *	p*K*_Py3CA_ ± *s *	p*K*_AA_ ± *s *
				or p*K*_3MePy_

ASA + Py3CA + AA	3.66 ± 0.05	—	5.02 ± 0.04	4.15 ± 0.05
ASA + 2NH_2_Py + 3MePy	3.61 ± 0.03	7.02 ± 0.03	—	5.82 ± 0.03

^*^p*K*_a_ value const. from the literature [see Table 11].

**Table 6:** The p*K*_a_'s values of compounds determined in the mixture containing four-weak electrolytes (acids and bases) and a strong acid (HCl) at 25°C.

Composition of D	p*K*_*n*_	p*K*_PhA_ ± *s*	p*K*_MA_ ± *s*	p*K*_Py3CA_ ± *s*	p*K*_A_ ± s
PhA + MA + Py3CA + A + HCl	p*K*_1_	3.11 ± 0.05	3.74 ± 0.03	4.8^*^	4.68 ± 0.02
	p*K*_2_	5.62 ± 0.03	—	—	—

Composition of D	p*K*_*n*_	p*K*_AA_ ± *s*	p*K*_Bi_ ± *s*	p*K*_ImH_ ± *s*	p*K*_BtH_ ± *s*

AA + Bi + ImH + BtH + HCl	p*K*_1_	4.29 ± 0.13	5.98 ± 0.14	7.55 ± 0.14	9.19 ± 0.14

^*^p*K*_a_ value const. from the literature [see Table 11].

**Table 7:** The p*K*_a_'s values of compounds determined in the multicomponent mixture of amino acid, heterocyclic moiety, and weak (Mes) and strong (HCl) acids at 25°C.

Composition of D	p*K*_*n*_	p*K*_His_ ± *s*	p*K*_Py3CA_	p*K*_Mes_	p*K*_ImH_
L-His	p*K*_3_	9.67 ± 0.01	—	—	—
L-His + HCl (1 : 1)	p*K*_2_	6.28 ± 0.01	—	—	—
	p*K*_3_	9.97 ± 0.01	—	—	—
L-His + HCl (1 : 2)	p*K*_1_	1.54 ± 0.04	—	—	—
	p*K*_2_	6.26 ± 0.01	—	—	—
	p*K*_2_	9.66 ± 0.01	—	—	—
L-His + Py3CA+ + Mes + ImH	p*K*_1_	—	—	6.28^*^	7.56^*^
	p*K*_2_	6.28^*^	4.68 ± 0.24	—	—
	p*K*_3_	10.06 ± 0.55	—	—	—
L-His + Py3CA + + Mes + ImH + HCl	p*K*_1_	—	4.81 ± 0.28	6.07 ± 0.14	7.49 ± 0.05
	p*K*_2_	6.43 ± 0.74	—	—	—
	p*K*_3_	10.06 ± 0.37	—	—	—

Composition of D	p*K*_*n*_	p*K*_Ala_	—	—	—

L-Ala	p*K*_2_	10.30 ± 0.01	—	—	—
L-Ala + HCl (1 : 1)	p*K*_1_	2.25 ± 0.01	—	—	—
	p*K*_2_	10.25 (0.07)	—	—	—

^*^p*K*_a_ value const. from the literature [see Table 11].

**Table 8:** The p*K*_a_'s values of compounds determined in the multicomponent mixture of phenol and enol OH–acids together with other weak electrolytes at 25°C.

Composition of D	p*K*_A_ ± *s*	p*K*_FA_ ± *s*	p*K*_4NO_2_PhOH_ ± *s*	—
A + FA + 4NO_2_PhOH	4.87 ± 0.02	3.05 ± 0.03 4.06 ± 0.03	7.42 ± 0.04	—

Composition of D	p*K*_MAL_ ± *s*	p*K*_FA_ ± *s*	p*K*_4NO_2_PhOH_ ± *s*	—

FA + MAL + 4NO_2_PhOH	3.59 ± 0.04 5.14 ± 0.02	2.50 ± 0.04 4.06 ± 0.04	7.4 ± 0.03	—

Composition of D	p*K*_AA_ ± *s*	p*K*_Mes_ ± *s*	p*K*_Pcm_ ± *s*	—

AA + Mes + Pcm	4.14 ± 0.03	6.24 ± 0.04	9.95 ± 0.05	—

Composition of D	p*K*_ASA_ ± *s*	p*K*_FA_ ± *s*	p*K*_4NO_2_PhOH_ ± *s*	p*K*_Kpf_

ASA + FA + 4NO_2_PhOH + Kpf	3.77 ± 0.05	2.91 ± 0.06 4.75 ± 0.37	7.52 ± 0.05	4.68 ± 0.36

**Table 9:** The p*K*_a_'s values of compounds determined in the multicomponent mixture which comprises barbituric acid, 2(1H)-pyrazylidene acetonitrile, and phthalic acid at 25°C.

Composition of D	p*K*_*n*_	p*K*_2(1H)PyAN_ ± *s*	p*K*_BA_ ± *s*	p*K*_PhA_ ± *s*
2(1H)PyAN + BA + PhA	p*K*_1_	7.10 ± 0.09	4.01 ± 0.04	2.39 ± 0.08
	p*K*_2_	—	—	5.76 ± 0.05

**Table 10:** The p*K*_a_'s values of compounds with pharmaceutical importance determined in the mixture containing other weak electrolytes 25°C.

Composition of D	p*K*_AA_ ± *s*	p*K*_Mes_ ± *s*	p*K*_Pcm_ ± *s*	p*K*_ASA_	p*K*_CA_
AA + Mes + Pcm	4.14 ± 0.03	6.24 ± 0.04	9.95 ± 0.05	—	—

Composition of D	p*K*_ASA_ ± *s*	p*K*_ImH_ ± *s*	p*K*_Eph_ ± *s*	—	—

ASA + ImH + Eph ∗ HCl	3.49 ± 0.03	7.21 ± 0.06	9.94 ± 0.05	—	—

Composition of D	p*K*_Met_ ± *s*	p*K*_ImH_ ± *s*	p*K*_Mes_ ± *s*	—	—

Met + ImH + Mes	2.28 ± 0.04	7.35 ± 0.02	6.19 ± 0.01	—	—

Composition of D	p*K*_ASA_ ± *s*	p*K*_Mes_ ± *s*	p*K*_L-His_ ± s	—	—

ASA + Mes + L-His	3.57 ± 0.03	6.29 ± 0.03	p*K*_1L-His_ = 1.54^*^ p*K*_2L-His_ = 5.96 ± 0.05 p*K*_3L-His_ = 9.67 ± 0.59	—	—

Composition of D	p*K*_KTL_ ± *s*	p*K*_Mes_ ± *s*	p*K*_L-His_ ± *s *	—	—

KTL + Mes + L-His	p*K*_1_ 3.09 ± 0.05 p*K*_2_ 6.15^*^	6.15^*^	p*K*_2L-His_ = 6.15^*^ p*K*_3L-His_ = 9.58 ± 0.06	—	—

Composition of D	p*K*_AA_ ± *s*	p*K*_Mes_ ± *s*	p*K*_Ppv_ ± *s *	p*K*_ASA_	p*K*_CA_

AA + Mes + Ppv	4.24 ± 0.02	6.55 ± 0.03	6.02 ± 0.06	—	—
AA + Mes + ASA + CA	4.05 ± 0.16	—	—	3.90 ± 0.16	p*K*_1_ = 2.53 ± 0.30 p*K*_2_ = 5.03 ± 0.33 p*K*_3_ = 6.5 ± 0.34

^*^p*K*_a_ value const. from the literature [see Table 11].

**Table 11:** Experimental and literature p*K*_a_'s values of the compounds under study.

No.	Compounds	p*K*_exp⁡_^*^	p*K*_literature_	Reference
1	A	4.81	4.55–4.78	[11]
2	AA	4.17	4.10	[15]
3	ASA	3.67	3.30–3.74	[16]
4	BA	4.01	4.02	[17]
5	Bi	5.98	5.66	[18]
6	CA	2.53 5.03 6.50	3.13 4.76 6.40	[19]
7	Eph	9.94	9.56	[20]
8	FA	2.88 4.28	3.02 4.39	[21]
9	Im	7.43	6.95	[22]
10	KTL	3.09 6.15	2.90–3.29 6.39	[16]
11	Ktp	4.68	4.6	[23]
12	L-ala	2.25 10.28	2.26–2.54 9.7–9.9	[18]
13	L-his	1.67 6.22 9.79	1.54 6.07 9.34	[22]
14	MA	3.48	3.18–3.41	[18]
15	3-MePy	5.82	5.61–6.02	[18]
16	MAL	3.59 5.14	3.11–3.30 4.46–5.12	[18]
17	Mes	6.27	6.27	[22]
18	Met	2.28	2.38	[16]
19	2-NH_2_Py	7.04	6.72–6.76	[16]
20	4-NH_2_Py	9.43	9.02–9.29	[16]
21	4-NO_2_PhOH	7.44	7.02–7.15	[11]
22	PhA	2.86 5.53	2.95 5.41	[22]
23	Ppv	6.02	6.21–6.49	[16]
24	2(1H)PyAN	7.10	—	—
25	Py-3CA	4.85	4.82	[24]
26	Py-4CA	4.72	4.84	[24]
27	2.6-PyDCA	2.41 4.72	2.00–3.45 4.47–4.87	[18]

^*^p*K*_exp_ calculated as ∑p*K*_*j*_/*n*.

## Abbreviations

*C*_0_: Concentration [mol/L] of weak electrolyte
D: Titrand (solution titrated)
T: Titrant (titrating solution)
*K*: Dissociation constant for a weak electrolyte
p*K* = −log⁡⁡*K*: Acidity parameter
*V*: Volume [mL] of T
*V*_0_: Volume [mL] of D
A: Aniline, aminobenzene (Sigma Aldrich > 99.5%)
AA: Ascorbic acid L(+), (5*R*)-[(1*S*)-1,2-dihydroxyethyl]-3,4-dihydroxyfuran-2(5*H*)-one (Chempur, p.a.)
ASA: Acetylsalicylic acid, 2-acetoxybenzoic acid (Sigma Aldrich, >99.5%)
BA: Barbituric acid, pyrimidine-2,4,6(1*H*,3*H*,5*H*)-trione (Fluka, p.a.)
Bi: 1*H* -Benzimidazole (Sigma Aldrich)
Bt: 1,2,3-Benzotriazole (Sigma Aldrich)
CA: Citric acid monohydrate, 2-hydroxypropane-1,2,3-tricarboxylic acid (P.P.H. Standard S. z o.o., p.a.)
Eph: Ephedrine hydrochloride, (R,S)-2-(methylamino)-1-phenylpropan-1-ol
FA: Fumaric acid, (*E*)-butenedioic acid (Sigma Aldrich, >99%)
Im: 1*H* -Imidazole (Sigma Aldrich)
Kpf: Ketoprofen, (*RS*)-2-(3-benzoylphenyl)propanoic acid
KTL: Ketoconazole, 1-[4-(4-{[(2*R*,4*S*)-2-(2,4-dichlorophenyl)-2-(1*H* -imidazol-1-ylmethyl)-1,3-dioxolan-4-yl]methoxy}phenyl)piperazin-1-yl]ethan-1-one
L-ala: L-Alanine, 2-aminopropanoic acid (Fluka)
L-his: L-Histidine, 2-amino-3-(1*H*-imidazol-4-yl)propanoic acid (Sigma Aldrich)
MA: Mandelic acid, 2-hydroxy-2-phenylacetic acid (Alfa Aesar Gmbh & Co, >99%)
MAL: Malic acid (hydroxybutanedioic acid)
Met: Metronidazole (2-(2-methyl-5-nitro-1*H* -imidazol-1-yl)ethanol)
3-MePy: 3-Methylpyridine (Sigma Aldrich)
Mes: 2-(N-Morpholino)ethanesulfonic acid hydrate (Sigma Aldrich, ≥99%)
2-NH_2_Py: 2-Aminopyridine (Sigma Aldrich)
4-NH_2_Py: 4-Aminopyridine (Sigma Aldrich)
4-NO_2_PhOH: 4-Nitrophenol (POCH S.A.)
Ppv: Papaverine (1-(3,4-dimethoxybenzyl)-6,7-dimethoxyisoquinoline)
Pcm: Paracetamol (*N*-(4-hydroxyphenyl)acetamide)
PhA: Phthalic acid, benzene-1,2-dicarboxylic acid (POCH S.A.)
2(1H)PyAN: *α*-2(1-H)pyrazylidene *α*-2-cyanoethylacetate
Py-3CA: Pyridine-3-carboxylic acid (Sigma Aldrich, p.a.)
Py-4CA: Pyridine- 4-carboxylic acid (Sigma Aldrich, p.a.)
2,6-PyDCA: Pyridine-2,6-dicarboxylic acid (Sigma Aldrich, p.a.).
